# A Case Report of Postpartum Hemorrhage Secondary to Snake Bite Envenomation: A Pathogenesis and Current Management Review

**DOI:** 10.1155/crog/1956143

**Published:** 2026-02-25

**Authors:** Felipe Loza-Hernandez, Alexis Haro, Gabriela Carrión, Homero Loza

**Affiliations:** ^1^ Faculty of Medical, Health and Life Sciences, International University of Ecuador, UIDE, Quito, Ecuador; ^2^ Obstetrics and Gynecology, Hospital Metropolitano, Quito, Ecuador, hospitalmetropolitano.org; ^3^ Obstetrics and Gynecology, Axxis Hospital, Quito, Ecuador; ^4^ Obstetrics and Gynecology, Hospital General Dr.Enrique Ortega Moreira, Guayaquil, Ecuador; ^5^ Obstetrics and Gynecology, Universidad Espíritu Santo (UEES) Clinic, Guayaquil, Ecuador; ^6^ Obstetrics and Gynecology, Hospital de los Valles, Quito, Ecuador, hospitaldelosvalles.com

**Keywords:** antivenom, case report, envenomation, postpartum hemorrhage, pregnancy, snake bite

## Abstract

**Background:**

Snakebite envenomation (SBE) is a tropical disease with significant global morbidity and mortality, particularly affecting low‐resource settings. During pregnancy, SBE poses unique challenges, increasing both maternal and fetal mortality. Limited access to antivenom and delayed treatment further worsen outcomes.

**Case Presentation:**

We report the case of a 36‐year‐old pregnant woman at 40.6 weeks gestation who presented to a rural Ecuadorian health center following a *Bothrops* snakebite to the right hand. Coagulopathy was detected and initially managed with antivenom. Because of fetal distress and oligohydramnios, an urgent cesarean section was performed. The patient subsequently developed severe postpartum hemorrhage secondary to venom‐induced coagulopathy (VICC), requiring subtotal hysterectomy, intensive care, and further antivenom administration. Postoperative recovery was favorable, and the patient was discharged in stable condition.

**Discussion:**

This case illustrates the complex pathophysiology of viper envenomation, including systemic coagulopathy, uterine atony, and thrombin‐induced postpartum hemorrhage. It highlights the diagnostic challenges, the limitations of standard coagulation tests in VICC, and the importance of early antivenom therapy. Delayed treatment in rural areas contributes significantly to maternal‐fetal morbidity. Obstetric complications such as placental abruption, fetal hypoxia, and hemorrhage must be anticipated in SBE during pregnancy.

**Conclusion:**

Timely antivenom administration and multidisciplinary management are essential to improve outcomes in pregnant patients with SBE. This case emphasizes the need for increased access to antivenom, improved diagnostic tools, and specialized obstetric care in resource‐limited regions affected by venomous snakebites.

## 1. Introduction

Snakebite envenomation (SBE) remains a significant public health concern, particularly in tropical and subtropical regions. The World Health Organization (WHO) estimates that 5.4 million snakebites occur annually, leading to 1.8–2.7 million cases of envenomation and approximately 81,000–138,000 deaths worldwide. The highest mortality rates are reported in Asia (57,000–100,000 deaths per year), Africa (20,000–32,000 deaths per year), and Latin America (3400–5000 deaths per year) [1]. In South America, Brazil is the most affected country, followed by Venezuela, Colombia, Peru, and Ecuador, where agricultural and rural workers are particularly vulnerable [2–6].

Among the most clinically relevant venomous snakes, species from the Viperidae family, particularly *Bothrops*, are responsible for most SBE cases in South America [7, 8]. The most common victims are adult males, often affected on the lower limbs or hands while working in agricultural settings [7, 8]. In addition to its direct health impact, SBE results in economic burdens due to loss of productivity and long‐term complications such as chronic kidney disease and psychological disorders [9].

Snakebites during pregnancy pose an even greater risk, with maternal mortality rates reported at 4.2% and fetal mortality rates reaching 19.2% [10]. The delay in medical care significantly influences outcomes, emphasizing the urgent need for accessible healthcare in rural areas [11]. SBE‐related maternal complications include placental abruption, preterm labor, and postpartum hemorrhage, while fetal risks include hypoxia, congenital abnormalities, and stillbirth [12].

Other sources estimate that the risk of fetal and neonatal death doubles after a snakebite (OR 2.17 and 2.79, respectively) [11]. One of the most important factors influencing maternal‐fetal mortality is the time elapsed from the snakebite to treatment, which accentuates the need for access to emergency care and timely antivenom delivery in rural and remote regions where most snakebites occur, rather than primary care availability itself. Addressing the burden of SBE requires improved access to antivenom therapy, early diagnostic tools, and public health strategies to mitigate exposure in high‐risk populations.

Given the burden of SBE in Latin America, it is important to present a clinical case from a developing country where access to healthcare, particularly in rural areas, remains a significant challenge. Case reports from such regions provide valuable insight into the real‐world implications of snakebites, highlighting barriers to timely diagnosis and treatment, the socioeconomic impact on affected individuals, and the necessity for improved healthcare infrastructure and antivenom availability. Presenting a case from Ecuador contributes to the understanding of SBE management in resource‐limited settings and emphasizes the urgency of addressing this neglected public health issue.

## 2. Methods and Ethics

The study was approved by the Ethics Committee of the General Dr. Enrique Ortega Moreira Hospital. As this report describes a single anonymized clinical case, formal Institutional Review Board (IRB) protocol registration was not required. Written informed consent was obtained from the patient for the use of clinical data and images for publication. The authors declare no conflicts of interest regarding the study and publication of this case report. Furthermore, the authors received no specific funding for the conduct of this work; university‐based financial support is being requested solely to cover the article processing charges (APCs).

## 3. Case Report

**Figure 1:**
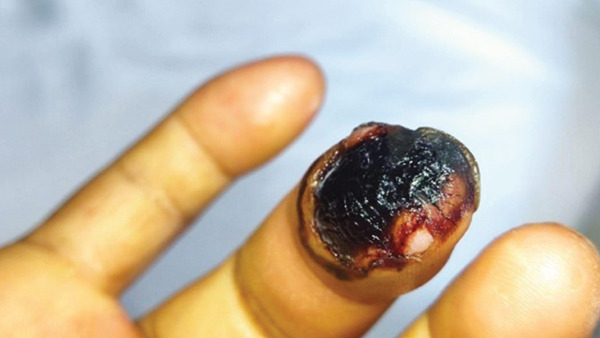
Snakebite on the right ring finger.

A 36‐year‐old female at 40.6 weeks of gestation presented to the La Maná Health Center approximately 30 min after sustaining a Bothrops snakebite on her right ring finger (Figure 1). Initial laboratory studies revealed hemoglobin of 12.1 g/dL, a white blood cell count of 13,800/*μ*L, and thrombocytopenia (92,000/*μ*L). Coagulation profiles were consistent with venom‐induced consumptive coagulopathy (VICC), showing a prolonged prothrombin time (PT 16.9 s, INR 1.7), prolonged activated partial thromboplastin time (aPTT 46 s), hypofibrinogenemia (140 mg/dL), and markedly elevated D‐dimer (5.1 *μ*g/mL). Renal function remained preserved, though liver enzymes were mildly elevated. The patient received four vials of polyvalent antivenom within 40 min of presentation before being transferred to a higher‐level facility for further management.

Upon arrival, fetal monitoring indicated a Category II tracing with decreased variability and oligohydramnios. An urgent segmental cesarean section was performed, during which scant amniotic fluid with a “pea soup” appearance was noted. Following delivery, the patient experienced uterine atony, which was initially managed using the B‐Lynch technique, uterotonic agents, and activation of the red code protocol (hemorrhage).

**Figure 2:**
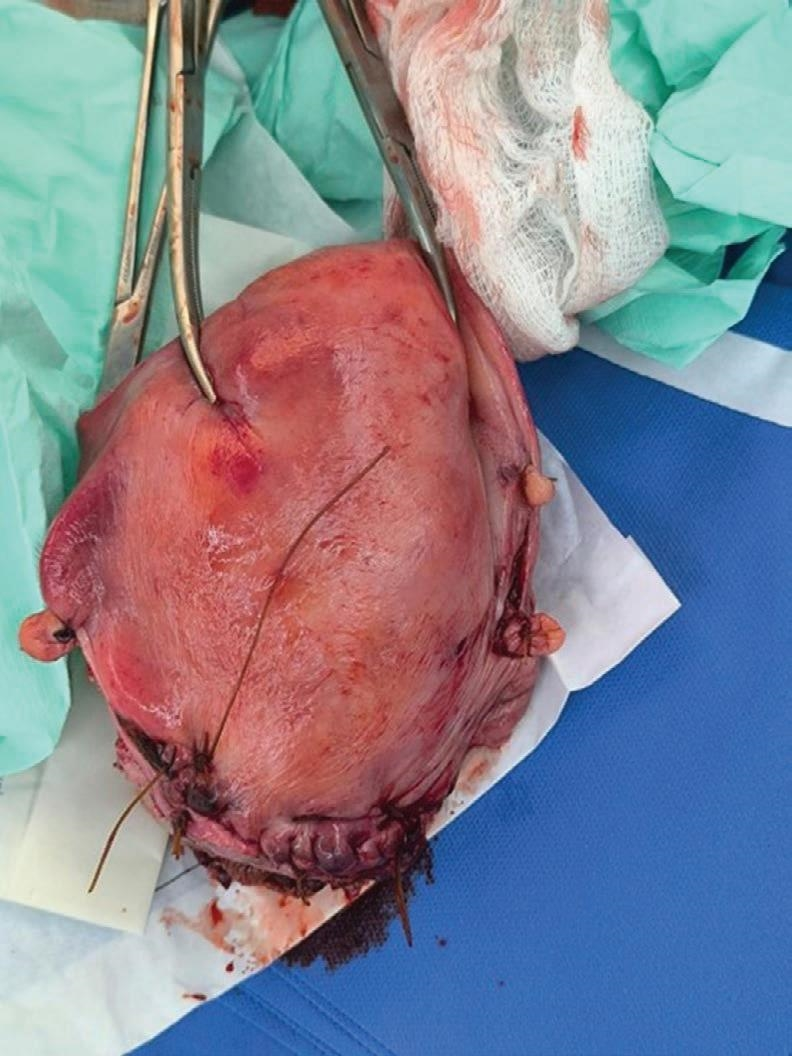
Post‐subtotal hysterectomy uterus.

Postoperatively, the patient was admitted to the intensive care unit (ICU) for mechanical ventilation and vasopressor support. Three hours post‐surgery, she suffered a secondary hemorrhage of 1000 mL due to persistent uterine atony, necessitating an emergency subtotal hysterectomy (Figure 2). To address the ongoing severe coagulopathy, 12 additional vials of antivenom were administered.

By the fourth ICU day, the patient’s condition improved, allowing for successful extubation and weaning to a nasal cannula. Vascular surgery was consulted for necrotic changes on the middle and distal phalanx of the right ring finger, recommending plastic surgery evaluation. Plastic surgeons performed hematoma drainage and debridement of hyperkeratosis in the palmar region.

By the seventh day, the patient was transferred to the gynecology ward due to significant clinical improvement, and by the ninth day, she was discharged in stable condition with scheduled follow‐ups for plastic surgery and psychological support.

## 4. Discussion

### 4.1. Epidemiology

The World Health Organization (WHO) estimates that 5.4 million snakebites occur annually [1]. On a global scale, SBE results in significant morbidity, with cases ranging between 1.8 and 2.7 million each year, and mortality rates estimated at 81,000–138,000 deaths predominantly in Asia (57,000–100,000 annual deaths), Africa (20,000–32,000 annual deaths), and Latin America (3400–5000 annual deaths) [1, 13]. Given that venomous snakes thrive in warm, humid environments, tropical South American countries are the most affected; Brazil reports the highest incidence, followed by Venezuela, Colombia, Peru, and Ecuador.

In the Amazonian region, SBE is primarily an occupational hazard [9]. Published literature from Colombia, Guatemala, and Ecuador consistently show that the most vulnerable populations are those in rural areas with low socioeconomic status, particularly agricultural workers [2–6]. Adult males are affected twice as often as females, with injuries most frequently occurring on the lower limbs or on the upper limbs in the attempt of defense. In this region, the most common snake that causes SBE corresponds to the Viperidae family, specifically the Bothrops type [7, 8].

Beyond mortality, SBE imposes a greater burden affecting the economic workforce and long‐term public health. Survivors have a higher morbidity due to local tissue damage, metabolic imbalances and renal long‐term injury [9]. Furthermore, a significant psychological impact has been documented, with survivors showing a three‐fold increase in depressive disorders compared with the general population [14].

Specifically referring to pregnant population, a literature review published in 2010 that studied the available data on PubMed, reported a maternal fatality rate secondary to SBE of 4.2% and a fetal mortality rate of 19.2% [10]. Other studies estimate that the risk of fetal and neonatal death duplicates after a snakebite (OR 2.17 and 2.79, respectively) [11]. A critical determinant of these outcomes is the time elapsed from the bite to treatment, underscoring the vital need for improved access to emergency care and antivenom in remote rural regions.

### 4.2. Physiopathology

Gutierrez et al. conducted a historical review of the evolution of the medical comprehension of snake venom toxicity [15]. In 1886, J.B. de Lacerda described hemorrhage in various organs such as muscle, abdominal organs, and the brain secondary to *Bothrops* snake bites. His histopathological description reports (translated from the original French) that *“the subcutaneous cellular tissue and the subjacent muscle were infiltrated with black irregular stains, dispersed violet spots and numerous bloody extravasations, offering a dark gelatin-like aspect,* partially coagulated” [16]. Nowadays, although we still have gaps in our knowledge, we understand the main mechanisms of snake venom toxicity.

Snakes have developed a complex system that allows them to produce venom, a modified form of saliva made of both toxic and non‐toxic proteins, peptides, and metal ions [9]. Venom is produced in glands and is secreted through fangs to be directly injected into the victim, enhancing the snake’s capacity to hunt and defend their natural habitat [2]. Snake venom is a complex biological mixture of toxic and non‐toxic proteins, peptides, and metal ions. In the case of Bothrops, the venom contains multiple toxins that act synergistically to disrupt hematologic physiology, primarily through the pathological activation or deactivation of coagulation factors within the common pathway of the coagulation cascade [17, 18].

First, phospholipase A2 (PLA2) catalyzes the breakdown of biological membranes through the disintegration of glycerophospholipids, causing hemolysis, myotoxicity, necrosis, and alteration of platelet aggregation [9]. Snake venom metalloproteases (SVMPs) are also hemotoxic. These enzymes paradoxically activate both procoagulants (such as prothrombin and Factor X), and anticoagulants pathways (fibrinolysis) [17, 19]. SVMPs also degrade components of endothelial basement membranes such as laminin, nidogen, fibronectin, collagen type IV, and proteoglycan, thereby disrupting vessel wall integrity causing bleeding and diffuse consumption of coagulation factors [15]. SVMP enzymes also affect the muscle fibers of mammals by collagenolytic activity that has been proven to delay or even prevent muscle fibers adequate regeneration causing nonreversible muscle damage [20]. Finally, snake venom serine proteases (SVSPs) (also known as *thrombin-like* enzymes) affect hemostasis mainly through cleavage of fibrinogen. In a physiological setting, this would emulate thrombin catalysis of fibrinogen into fibrin to stabilize the forming clot. However, in an SBE context, SVSPs cause a useless consumption of fibrinogen, thus preventing optimal coagulation.

The outcome of these molecular mechanisms is the diffuse and disorganized formation of microthrombi. These small clots translate into microangiopathy that directly affects the muscles the kidneys, which are the main end‐organ site of damage causing acute kidney injury (AKI) [21, 22]. Therefore, renal injury can be explained by two mechanisms: first renal hypo‐perfusion due to hypovolemia or thrombosis and, second, due to vascular endothelial injury caused directly by the snake toxins or indirectly by circulating substances such as myoglobin or other products of hemolysis, finally causing renal tubular and cortical necrosis [23].

### 4.3. Diagnosis

In clinical practice, several authors have offered algorithms and strategies to manage snakebites. The first step is always to identify the type of SBE, an appropriate clinical history should try to identify the species of the snake. Epidemiologically, Viperidae species (more common in South America) cause hemo‐toxicity and Elapidae species (more common in Asia and Africa) are neurotoxic causing paralysis and respiratory failure [1, 13, 24]. Secondly, the health provider should diagnose and accurately assess the severity of the envenomation to prevent or anticipate the most important systemic effects such as (in case of viper snake bite) spontaneous hemorrhage, disseminated intravascular coagulation, cerebral hemorrhage, AKI, and cardiovascular shock [25].

The initial anamnesis should include the anatomical part that was bitten, the time elapsed since the accident, the appearance of the snake and current symptoms [13]. The first signs after the bite are local pain, edema, blistering, redness, bruising, and bleeding. Later signs can include neurological deterioration or dark‐colored urine (representing cerebral hemorrhage or AKI) [26]. Laboratory tests should be taken including complete blood count, a peripheral blood smear, coagulation tests, urinalysis, electrolytes, and arterial or venous gasometer [24, 25, 27]. The goal of these tests is to determine the current state of the SBE and assess the severity and the possible complications.

It is important for the practitioner to know that not every coagulation test available is useful for SBE. Currently, the importance of early, affordable, and effective diagnosis of venom‐induced consumption coagulopathy (VICC) is critical. A study published in 2024 compared different commercial coagulation tests available to try to identify the most promising devices for future clinical validation [18]. Point‐of‐care devices were reviewed for measuring INR, Activated Clotting Time (ACT), aPTT, fibrinogen, and fibrin degradation products (D‐dimer and FDPs) [18]. The study concluded that conventional PT, aPTT, and INR tests may be unreliable because they depend on the conversion of fibrinogen to fibrin to form a stable clot. However, in severe VICC, fibrinogen is so depleted that these tests may result in “infinite” readings or, paradoxically, appear normal if the reagents used are thrombin independent. Furthermore, fibrin degradation products such as D‐Dimer can be falsely low because they are only produced from stable clots. In VICC, fibrinogen is directly fragmented by the snake’s venom, and there is a diffuse formation of unstable clots. For bedside monitoring in resource‐limited settings, tests that are recommended are the ones that measure fibrin (fibrin‐depending INR such as Hemochron Signature Elite [Accriva Diagnostics], microINR [iLine Microsystems], Coag‐Sense [CoaguSense Inc.]) and fibrinogen measurement. A more affordable bedside option that is still reliable is the ACT. This test measures the time it takes for whole blood to clot after activation with celite or kaolin (if it takes more than 130 s, the test is positive and if it takes longer than 180–240 s, it can be classified as a severe VICC). It provides a quicker and standardized assessment of coagulation function, making it more reliable than the 20‐min Whole Blood Clotting Test (20WBCT) that can be altered by the test‐tube quality [18].

Given that a recent Australian systematic review found AKI in 94% of snakebite‐associated thrombotic microangiopathy (TMA) cases, the diagnosis of TMA should be made from the very beginning of the evaluation [21]. TMA can be defined by the schistocyte quantitation in a peripheral blood smear: A schistocyte count of ≥ 1.0% on a peripheral blood smear is a highly sensitive (90%) and specific (71%) marker for predicting AKI in patients with SBE [21, 28].

In pregnant patients, given the high rates of fetal loss secondary to SBE, fetal well‐being should be assessed as soon as possible by an experienced practitioner [29]. Several mechanisms explaining the negative impact on pregnancy have been identified, including direct effect of the venom passing through the placenta, fetal hypoxia secondary to maternal shock, placental bleeding, abruptio placentae, venom‐induced uterine contractions and hyperpyrexia, and hyper‐cytokine release due to tissue damage [10, 12].

Snakebite accidents have been reported in every trimester of pregnancy, each with an even risk of loss [10]. Although the cause is not always specified, abruptio placentae is one of the most frequent complications secondary to SBE. Malformations including hydrocephalus, polydactyly, and intracranial hemorrhage were reported in 0.14% of cases (*n* = 3 of 213) studied by Langley in 2010. Another study from Brazil reported 274 cases of snakebites in pregnant patients with high rates of fetal and neonatal fatality associated with delayed access to treatment [11]. Fetal malformations following SBE are rare but biologically plausible. Proposed mechanisms include maternal systemic inflammatory response that would disrupt early organogenesis and transplacental transfer of venom components, such as metalloproteinases and phospholipase A2, which may induce fetal tissue injury and intracranial hemorrhage, as described in isolated case reports [10]. These mechanisms suggest that fetal abnormalities are more likely the result of indirect maternal–placental physiological disturbances rather than a true direct teratogenic effect of the venom.

The search of the terms “(Snakebite) AND (postpartum hemorrhage)” in PubMed only yields one case report of an 8‐month pregnant 30‐year‐old woman from Burkina Faso that suffered a Viper snakebite in the ankle [30]. In this case, labor started spontaneously 48 h later. A relative of the patient attended the birth and tried to take her to a medical assistant nearby; however, the woman died during transportation 4 h after delivery due to massive hemorrhage. The newborn died by Day 8, the cause is not specified. In fact, the case presented by this study is quite unique because SBE caused a post‐partum hemorrhage, a rare complication secondary to VICC (that required progressive management until the final hysterectomy). This corresponds to the “Thrombin” mechanism of postpartum hemorrhage, the less frequent of the 4 Ts: tone (uterine atony), trauma (lacerations or uterine rupture), tissue (retained placenta or clots), and thrombin (clotting‐factor deficiency) [31].

### 4.4. Management

Initial management of SBE, must prioritize hemodynamic stabilization through adequate hydration and close monitoring of blood pressure and urine output to mitigate the risks of hypovolemia and AKI [27]. The bite injury should be regularly monitored to prevent severe edema and compartmental syndrome [12, 27].

Apart from the obstetric individualized management, SBE in a pregnant patient should be managed following the same principles that in non‐pregnant population. Antisnake‐venom (ASV) should be the cornerstone of treatment and should be given to any snakebite victim with progressive signs and symptoms. In pregnant patients, the evidence indicates that delayed access to ASV significantly increases fetal mortality [11]. While there is no universal standard dose, it is estimated that 10 mL of polyvalent ASV neutralizes approximately 6 mg of venom. Considering that an average viper bite injects roughly 63 mg, substantial dosing is often required [32]. It should be taken into consideration that ASV can only prevent the effect of circulating venom that has not yet bound to cellular receptors [33]. After administration, systemic bleeding typically stops within 30 min, and the coagulation profile improves within 6 h [29]. Antibiotic coverage should be individualized considering the elevated risk of local infection in the wound due to the micro‐organisms present in the snake’s mouth.

ASV is far from a perfect treatment: there is a high risk of anaphylaxis and severe hypersensitivity reactions [34]. Plus, the production and care of ASV is difficult and expensive because it relies on the hyper‐immunization of mammals like horses, donkeys, and sheep. This means that there is a high risk for caretakers that must inject low doses of venom to the source‐mammal and to the later [27]. Furthermore, the requirement for a strict cold chain to maintain potency further complicates its availability in the remote, rural regions where it is most needed [35].

The reposition of coagulation factors has been proposed to compensate for VICC; however, the evidence is contradictory in this matter. Some studies have concluded that there is no improvement in the coagulation profile in patients treated with clotting factor replacement therapy after antivenom administration [33]. In fact, 7.8% of patients that received fresh frozen plasma (FFP) therapy from Zeng et al.′s trial developed anaphylaxis and/or heart failure. However, an Australian randomized controlled trial for the use of FFP for treating VICC reported that 6 h post‐antivenom administration, 73% of patients who received FFP achieved an INR < 2, compared with only 25% in the non‐FFP group [36]. An important result from this study is that early FFP administration (< 6–8 h post‐bite) may be less effective, supposedly due to the consumption of newly introduced clotting factors by the still circulating venom [36, 37].

A major issue in the management of snakebite accidents is the high cost of treatment, particularly for low‐income and rural populations, who are the most affected by this type of incident. It has been reported that the cost of treating a SBE in India can exceed twice the country’s GDP per capita, amounting to the equivalent of up to 10 years’ wages for an average farm worker [38]. This issue must be taken into consideration for clinical and research matters.

To address the limitations of animal‐derived antivenoms (including hypersensitivity reactions and the animal hyperimmunization process and risks), current research is focused on small‐molecule enzyme inhibitors. One example of these new treatments is Varespladib, a phospholipase A2 (PLA2) inhibitor, which has demonstrated effectiveness in treating envenomation‐induced coagulopathy caused by certain snake species [39, 40].

## 5. Conclusion

This case highlights the importance of early antivenom administration in mitigating the systemic effects of *Bothrops* envenomation, particularly coagulopathy. Delays in treatment can significantly increase maternal and fetal morbidity and mortality, emphasizing the need for accessible antivenom in rural and resource‐limited settings.

SBE during pregnancy necessitates seamless, coordinated care between obstetricians, intensivists, and surgical teams. As demonstrated in this report, viper envenomation can lead to severe complications, including uterine atony and life‐threatening postpartum hemorrhage, requiring aggressive management strategies such as the B‐Lynch technique, uterotonic therapy, and emergency hysterectomy. Additionally, fetal risks such as hypoxia and oligohydramnios reinforce the need for continuous fetal monitoring and rapid clinical decision‐making to optimize outcomes for both mother and child in these complex emergencies.

## Funding

The authors received no specific funding for the conduct of this work. University‐based financial support from Universidad Internacional Del Ecuador (UIDE) is being requested solely to cover the article processing charges.

## Conflicts of Interest

The authors declare no conflicts of interest.

## Data Availability

All data supporting the findings of this case report are contained within the manuscript. No additional datasets were generated or analyzed.

## References

[1] Organización Mundial de la Salud , Envenenamiento por mordedura de serpiente, 2023, Organización Mundial de la Salud, (accessed April 18, 2025) https://www.who.int/es/news-room/fact-sheets/detail/snakebite-envenoming.

[2] Redondo-Guerra J. and Muegues Á. , Vista de Mordeduras de serpientes en un área de explotación minera. Cesar, Colombia 2017-2019, Revista Agunkuyâa. (2020) 10, no. 2, 10.33132/27114260.1906.

[3] Márquez Gómez M. A. and Gómez Díaz G. M. , Ophidic Accident in the Department of Sucre Colombia, Nova. (2015) 13, 39–46.

[4] Guzmán Terán C. , Villa Dangond H. , and Calderón R. A. , Análisis epidemiológico y clínico de intoxicaciones agudas atendidas en Montería, Colombia, Revista Médica de Risaralda. (2015) 21, 17–21.

[5] Guerra J. R. and Muegues Á. A. , Mordeduras de serpientes en un área de explotación minera. Cesar, Colombia. 2017–2019, Revista Agunkuyâa. (2020) 10, no. 2, 10.33132/27114260.1906.

[6] Guerra-Centeno D. , Perfil epidemiológico del accidente ofídico en las tierras bajas de Guatemala, Ciencia, Tecnología Y Salud. (2016) 3, no. 2, 127–138, 10.36829/63CTS.V3I2.112.

[7] Roriz K. R. P. S. , Zaqueo K. D. , Setubal S. S. , Katsuragawa T. H. , da Silva R. R. , Fernandes C. F. C. , Cardoso L. A. P. , de S R. M. M. , Soares A. M. , Stábeli R. G. , and Zuliani J. P. , Epidemiological Study of Snakebite Cases in Brazilian Western Amazonia, Revista da Sociedade Brasileira de Medicina Tropical. (2018) 51, no. 3, 338–346, 10.1590/0037-8682-0489-2017, 2-s2.0-85049256671, 29972565. 29972565

[8] Monteiro W. M. , Contreras-Bernal J. C. , Bisneto P. F. , Sachett J. , Mendonça da Silva I. , Lacerda M. , Guimarães da Costa A. , Val F. , Brasileiro L. , Sartim M. A. , Silva-de-Oliveira S. , Bernarde P. S. , Kaefer I. L. , Grazziotin F. G. , Wen F. H. , and Moura-da-Silva A. M. , Bothrops atrox, The Most Important Snake Involved in Human Envenomings in the Amazon: How Venomics Contributes to the Knowledge of Snake Biology and Clinical Toxinology, Toxicon X. (2020) 6, 100037, 10.1016/J.TOXCX.2020.100037. 32550592 PMC7285970

[9] Williams H. F. , Layfield H. J. , Vallance T. , Patel K. , Bicknell A. B. , Trim S. A. , and Vaiyapuri S. , The Urgent Need to Develop Novel Strategies for the Diagnosis and Treatment of Snakebites, Toxins (Basel). (2019) 11, no. 6, 10.3390/TOXINS11060363, 2-s2.0-85068540712. PMC6628419 31226842

[10] Langley R. L. , Snakebite During Pregnancy: A Literature Review, Wilderness & Environmental Medicine. (2010) 21, 54–60, 10.1016/J.WEM.2009.12.025, 2-s2.0-77950159201. 20591355

[11] Nascimento T. P. , Vilhena Silva-Neto A. , Baia-Da-silva D. C. , da Silva Balieiro P. C. , Baleiro AA S. , Sachett J. , Brasileiro L. , Sartim M. A. , Martinezespinosa F. E. , Wen F. H. , Pucca M. B. , Gerardo C. J. , Sampaio V. S. , de Aquino P. F. , and Monteiro W. M. , Pregnancy Outcomes After Snakebite Envenomations: A Retrospective Cohort in the Brazilian Amazonia, PLoS Neglected Tropical Diseases. (2022) 16, no. 12, e0010963, 10.1371/JOURNAL.PNTD.0010963, 36469516. 36469516 PMC9754599

[12] Sebe A. , Satar S. , and Acikalin A. , Snakebite During Pregnancy, Human & Experimental Toxicology. (2005) 24, 341–345, 10.1191/0960327105HT535OA, 2-s2.0-23744497756. 16119247

[13] Gutiérrez J. M. , Calvete J. J. , Habib A. G. , Harrison R. A. , Williams D. J. , and Warrell D. A. , Snakebite Envenoming, Nature Reviews. Disease Primers. (2017) 3, 10.1038/NRDP.2017.63, 2-s2.0-85045233195. 28980622

[14] Williams S. S. , Wijesinghe C. A. , Jayamanne S. F. , Buckley N. A. , Dawson A. H. , Lalloo D. G. , and de Silva H. J. , Delayed Psychological Morbidity Associated With Snakebite Envenoming, PLoS Neglected Tropical Diseases. (2011) 5, no. 8, 10.1371/JOURNAL.PNTD.0001255, 2-s2.0-80052417658. PMC3149015 21829741

[15] Gutiérrez J. M. , Escalante T. , Rucavado A. , and Herrera C. , Hemorrhage Caused by Snake Venom Metalloproteinases: A Journey of Discovery and Understanding, Toxins (Basel). (2016) 8, no. 4, 10.3390/TOXINS8040093, 2-s2.0-84962321195. PMC4848620 27023608

[16] LaCerda J. , Leçons sur le Venin des Serpents du Brésil et sur la Méthode de Traitement des Morsures Venimeuses par le Permanganate de Potasse, 1884, Librairie Lombaerts.

[17] Xiong S. and Huang C. , Synergistic Strategies of Predominant Toxins in Snake Venoms, Toxicology Letters. (2018) 287, 142–154, 10.1016/J.TOXLET.2018.02.004, 2-s2.0-85042193389, 29428543. 29428543

[18] Abouyannis M. , Marriott A. E. , Stars E. , Kitchen D. P. , Kitchen S. , Woods T. A. L. , Kreuels B. , Amuasi J. H. , Monteiro W. M. , Stienstra Y. , Senthilkumaran S. , Isbister G. K. , Lalloo D. G. , Ainsworth S. , and Casewell N. R. , Handheld Point-of-Care Devices for Snakebite Coagulopathy: A Scoping Review, Thrombosis and Haemostasis. (2025) 125, no. 5, 405–420, 10.1055/A-2407-1400. 39214143 PMC12040437

[19] Ramos O. H. P. and Selistre-De-Araujo H. S. , Snake Venom Metalloproteases—Structure and Function of Catalytic and Disintegrin Domains, Comparative Biochemistry and Physiology Part C: Toxicology & Pharmacology. (2006) 142, 328–346, 10.1016/J.CBPC.2005.11.005. 16434235

[20] Williams H. F. , Mellows B. A. , Mitchell R. , Sfyri P. , Layfield H. J. , Salamah M. , Vaiyapuri R. , Collins-Hooper H. , Bicknell A. B. , Matsakas A. , Patel K. , and Vaiyapuri S. , Mechanisms Underpinning the Permanent Muscle Damage Induced by Snake Venom Metalloprotease, PLoS Neglected Tropical Diseases. (2019) 13, no. 1, e0007041, 10.1371/journal.pntd.0007041, 2-s2.0-85061237591. 30695027 PMC6368331

[21] Noutsos T. , Currie B. J. , Lek R. A. , and Isbister G. K. , Snakebite Associated Thrombotic Microangiopathy: A Systematic Review of Clinical Features, Outcomes, and Evidence for Interventions Including Plasmapheresis, PLoS Neglected Tropical Diseases. (2020) 14, no. 12, e0008936, 10.1371/JOURNAL.PNTD.0008936, 33290400. 33290400 PMC7748274

[22] Sonavane M. , Almeida J. R. , Rajan E. , Williams H. F. , Townsend F. , Cornish E. , Mitchell R. D. , Patel K. , and Vaiyapuri S. , Intramuscular Bleeding and Formation of Microthrombi During Skeletal Muscle Damage Caused by a Snake Venom Metalloprotease and a Cardiotoxin, Toxins (Basel). (2023) 15, no. 9, 10.3390/TOXINS15090530. PMC10536739 37755956

[23] Albuquerque P. L. , da Silva Junior G. B. , Meneses G. C. , Martins A. M. , Lima D. B. , Raubenheimer J. , Fathima S. , Buckley N. , and Daher E. D. , Acute Kidney Injury Induced by Bothrops Venom: Insights Into the Pathogenic Mechanisms, Toxins (Basel). (2019) 11, no. 3, 10.3390/toxins11030148, 2-s2.0-85062609142, 30841537. PMC6468763 30841537

[24] Noutsos T. , Currie B. J. , Wijewickrama E. S. , and Isbister G. K. , Snakebite Associated Thrombotic Microangiopathy and Recommendations for Clinical Practice, Practice. (2022) 14, no. 1, 10.3390/toxins14010057, 35051033. PMC8778654 35051033

[25] Albuquerque P. L. M. M. , Paiva J. H. H. G. L. , Martins A. M. C. , Meneses G. C. , Da Silva G. B. , Buckley N. , and Daher E. D. F. , Clinical Assessment and Pathophysiology of Bothrops Venom-Related Acute Kidney Injury: A Scoping Review, Journal of Venomous Animals and Toxins including Tropical Diseases. (2020) 26, e20190076, 10.1590/1678-9199-JVATITD-2019-0076, 32704246. 32704246 PMC7359628

[26] Torrez P. P. Q. , Said R. , Quiroga M. M. M. , Duarte M. R. , and França F. O. S. , Forest Pit Viper (Bothriopsis bilineata bilineata) Bite in the Brazilian Amazon With Acute Kidney Injury and Persistent Thrombocytopenia, Toxicon. (2014) 85, 27–30, 10.1016/J.TOXICON.2014.04.001, 2-s2.0-84899993682, 24726466. 24726466

[27] Maduwage K. and Isbister G. K. , Current Treatment for Venom-Induced Consumption Coagulopathy Resulting From Snakebite, PLoS Neglected Tropical Diseases. (2014) 8, no. 10, 10.1371/journal.pntd.0003220, 2-s2.0-84920531793. PMC4207661 25340841

[28] Noutsos T. , Currie B. J. , Isoardi K. Z. , Brown S. G. A. , and Isbister G. K. , Snakebite-Associated Thrombotic Microangiopathy: An Australian Prospective Cohort Study [ASP30], Clinical Toxicology. (2022) 60, no. 2, 205–213, 10.1080/15563650.2021.1948559. 34328386

[29] Singh S. and Mohanty R. R. , Vasculotoxic Snakebite Envenomation: Management Challenges in Pregnancy, Obstetric Medicine. (2021) 14, no. 3, 190–192, 10.1177/1753495X20952640, 34646350. 34646350 PMC8504311

[30] D’Ambruoso L. , Byass P. , and Ouedraogo M. , Maternal Death Due to Postpartum Hemorrhage After Snakebite, International Journal of Gynaecology and Obstetrics. (2008) 102, no. 1, 10.1016/J.IJGO.2008.03.006, 2-s2.0-46549088644, 18436221. 18436221

[31] Shields L. E. , Goffman D. , and Caughey A. B. , Practice Bulletin No. 183: Postpartum Hemorrhage, Obstetrics and Gynecology. (2017) 130, e168–e186, 10.1097/AOG.0000000000002351, 2-s2.0-85032013086. 28937571

[32] World Health Organization , Regional OFfice in South-East Asia. Guidelines for Management of Snakebites, 2016, 2nd edition, WHO Regional Office in SOuth-East Asia.

[33] Zeng L. , Liang Q. , Liang Z. , Han J. , Wu M. , Liu R. , and Wang X. , Effectiveness of Clotting Factor Replacement Therapy After Antivenom Treatment on Coagulopathic Envenomation Following Green Pit Viper Bites: A Retrospective Observational Study, BMC Emergency Medicine. (2022) 22, no. 1, 10.1186/S12873-022-00569-W. PMC8772100 35045831

[34] de Silva H. A. , Pathmeswaran A. , Ranasinha C. D. , Jayamanne S. , Samarakoon S. B. , Hittharage A. , Kalupahana R. , Ratnatilaka G. A. , Uluwatthage W. , Aronson J. K. , Armitage J. M. , Lalloo D. G. , and de Silva H. J. , Low-Dose Adrenaline, Promethazine, and Hydrocortisone in the Prevention of Acute Adverse Reactions to Antivenom Following Snakebite: A Randomised, Double-Blind, Placebo-Controlled Trial, PLoS medicine. (2011) 8, no. 5, e1000435, 10.1371/JOURNAL.PMED.1000435, 2-s2.0-79957979890, 21572992. 21572992 PMC3091849

[35] Rojas G. , Espinoza M. , Lomonte B. , and Gutiérrez J. , Effect of Storage Temperature on the Stability of the Liquid Polyvalent Antivenom Produced in Costa Rica, Toxicon. (1990) 28, no. 1, 101–105, 10.1016/0041-0101(90)90011-U, 2-s2.0-0025278038, 2330601. 2330601

[36] Isbister G. K. , Buckley N. A. , Page C. B. , Scorgie F. E. , Lincz L. F. , Seldon M. , Brown S. G. A. , and ASP Investigators , A Randomized Controlled Trial of Fresh Frozen Plasma for Treating Venom-Induced Consumption Coagulopathy in Cases of Australian Snakebite (ASP-18), Journal of Thrombosis and Haemostasis. (2013) 11, no. 7, 1310–1318, 10.1111/jth.12218, 2-s2.0-84880431779, 23565941. 23565941

[37] Berling I. and Isbister G. K. , Hematologic Effects and Complications of Snake Envenoming, Transfusion Medicine Reviews. (2015) 29, 82–89, 10.1016/J.TMRV.2014.09.005, 2-s2.0-84927037595. 25556574

[38] Vaiyapuri S. , Vaiyapuri R. , Ashokan R. , Ramasamy K. , Nattamaisundar K. , Jeyaraj A. , Chandran V. , Gajjeraman P. , Baksh M. F. , Gibbins J. M. , and Hutchinson E. G. , Snakebite and Its Socio-Economic Impact on the Rural Population of Tamil Nadu India, PloS one. (2013) 8, e80090, 10.1371/JOURNAL.PONE.0080090, 2-s2.0-84894272081. 24278244 PMC3836953

[39] Chowdhury A. , Youngman N. J. , Liu J. , Lewin M. R. , Carter R. W. , and Fry B. G. , The Relative Efficacy of Chemically Diverse Small-Molecule Enzyme-Inhibitors Against Anticoagulant Activities of Black Snake (Pseudechis spp.) Venoms, Toxicology Letters. (2022) 366, 26–32, 10.1016/J.TOXLET.2022.06.009, 35788045. 35788045

[40] Youngman N. J. , Lewin M. R. , Carter R. , Naude A. , and Fry B. G. , Efficacy and Limitations of Chemically Diverse Small-Molecule Enzyme-Inhibitors Against the Synergistic Coagulotoxic Activities of Bitis Viper Venoms, Molecules. (2022) 27, no. 5, 10.3390/MOLECULES27051733. PMC8911647 35268832

